# Anomalous heat transfer modes of nanofluids: a review based on statistical analysis

**DOI:** 10.1186/1556-276X-6-391

**Published:** 2011-05-19

**Authors:** Antonis Sergis, Yannis Hardalupas

**Affiliations:** 1 The Department of Mechanical Engineering, Imperial College London, London SW7 2AZ, UK

## Abstract

This paper contains the results of a concise statistical review analysis of a large amount of publications regarding the anomalous heat transfer modes of nanofluids. The application of nanofluids as coolants is a novel practise with no established physical foundations explaining the observed anomalous heat transfer. As a consequence, traditional methods of performing a literature review may not be adequate in presenting objectively the results representing the bulk of the available literature. The current literature review analysis aims to resolve the problems faced by researchers in the past by employing an unbiased statistical analysis to present and reveal the current trends and general belief of the scientific community regarding the anomalous heat transfer modes of nanofluids. The thermal performance analysis indicated that statistically there exists a variable enhancement for conduction, convection/mixed heat transfer, pool boiling heat transfer and critical heat flux modes. The most popular proposed mechanisms in the literature to explain heat transfer in nanofluids are revealed, as well as possible trends between nanofluid properties and thermal performance. The review also suggests future experimentation to provide more conclusive answers to the control mechanisms and influential parameters of heat transfer in nanofluids.

## Introduction

Nanofluids are fluids that contain small volumetric quantities (around 0.0001-10%) of nanosized suspensions of solid particles (100 nm and smaller in size). This kind of fluids exhibit anomalous heat transfer characteristics and their use as advanced coolants along with the benefits over their conventional counterparts (pure fluids or micron-sized suspensions/slurries) is investigated.

Nanofluids were invented by U.S. Choi of the Argonne National Laboratory (ANL) in 1993, during an investigation around new coolants and cooling technologies, as part of the "Advanced Fluids Program" project taking place At (ANL). The term "Nanofluids" was subsequently coined to this kind of colloidal suspensions by Choi in 1995 [1].

Since then, thriving research was undertaken to discover and understand the mechanisms of heat transfer in nanofluids. The knowledge of the physical mechanisms of heat transfer in nanofluids is of vital importance as it will enable the exploitation of their full heat transfer potential.

Several literature review papers were issued by researchers in the last years [2-6]. However, it is the current authors' belief that previous reviewers failed to present all the observations and results obtained from the literature in a clear and understanding method. The main problems arise from the fact that the application of nanofluids as coolants is a novel practise with no established physical foundations explaining the observed anomalous heat transfer characteristics. In addition, due to the recent growth of this area, there are no procedures to follow during testing for the evaluation of the thermal performance. As a consequence, traditional methods of performing a literature review may be inadequate in presenting an unbiased, objective and clear representation of the bulk of the available literature.

It was hence decided to perform a statistical analysis of the findings of the available publications in the literature in order to alleviate the problems faced by previous reviewers. The statistical analysis would enable the depiction of observations on comprehensive charts (histograms and scatter diagrams) hence making possible the extraction of conclusions in a more solid and mathematically trustworthy manner. The present literature review gives the same amount of weight to all of the observations available in the literature.

This review addresses the following questions:

a. What are the general heat transfer characteristics of nanofluids?

b. What are the trends linking the heat transfer performance of certain nanofluids with their by-part mixture parameters?

c. What are the most prevailing theories explaining the anomalous heat transfer behaviour observed in nanofluids?

The next section of this article describes the nanofluid characteristics followed by "Methodology of statistical analysis section". The next two sections present the results of the analysis obtained. "Nanoemulsions" section of this review contains brief information regarding a different type of fluids that has started emerging in the literature recently and might in the future be incorporated into the broader category of nanofluids. The final section contains the main conclusions reached by the current review.

## Characteristics of nanofluids

This section epitomizes the most common nanofluid preparation methods by providing information about the last stages of the fluid creation. Note that the "Quality" of a nanofluid represents the extent of achievability of the desired properties of the mixture.

The desired properties of a nanofluid are:

a. Even, durable and stable suspension of the solid nanoparticles in the host fluid (Basefluid)

b. Low or no formation of agglomerates

c. No chemical change of the basefluid (i.e. the solid particles must not chemically react with the host fluid).

Nanofluids follow either single or multi-step creation methods. The single-step creation approach refers to a direct evaporation method (Vacuum Evaporation onto a Running Oil Substrate-VEROS). This method attains the best quality nanofluids; however, there are substantial limitations on the flexibility to create customised nanoparticle volumetric concentrations and basefluid type samples.

The multi-step method provides more flexibility, but, in general, with a penalty in the quality of the attained mixture. Nanofluids can be created either by diluting a very dense solution of the required nanofluid with the matching basefluid or by mixing directly the nanoparticles of choice with the desired basefluid. The first procedure provides more flexibility than the single-step method as the nanoparticles' volumetric concentration can be made to order; however, the quality of the resulting nanofluid is lower than the one achieved via the single-step method.

The second approach of the multi-step method is the most widely used amongst researchers, since it provides maximum flexibility to control the volumetric concentration of the nanoparticles, along with the Basefluid type to be customised given the nanoparticle material, shape and size. On the other hand, this procedure delivered the lowest quality of nanofluids in comparison to all the other methods [1].

The most common liquids used as basefluid are conventional coolants, such as deionised water, engine oil, acetone, ethylene glycol. The most common nanoparticle materials used are aluminium (Al), aluminium oxide (Al_2_O_3_), copper (Cu), copper oxide (CuO), gold (Au), silver (Ag), silica dioxide (SiO_2_), titanium dioxide (TiO_2_) and carbon nanotubes (CNTs either single-walled, double-walled or multi-walled).

## Methodology of statistical analysis

In order to tackle the topics mentioned in "Introduction" section of this paper, the present researchers resolute to following a statistical investigation of a large sample of findings collected from the available literature.

The analysis was performed in three levels. The first level consists of the bulk of the findings from all the published work and enables the demonstration of a general view of the thermal performance of nanofluids. The second level focuses on the most commonly studied nanofluid types and compositions and makes possible to extract trends linking the various nanofluid properties with their thermal performance. The third and final level narrows the sample to include a selection of findings from simple geometry experiments (consisting of travelling hot wire and pipe flow type, instead of complex geometries), ignoring theoretical investigations, thus providing an insight into what appear to be the controlling parameters of thermal performance of nanofluids. Additionally, the final level of analysis reveals what is currently missing from the literature and indicates what aspects need to be investigated further to reach a more conclusive result regarding the links between thermal performance and nanofluid properties.

Findings were gathered regarding the observed enhancement for several heat transfer modes (conduction, convection, pool boiling and critical heat flux) compared to the heat transfer performance of the basefluid alone. Additional information was recorded linking the observed enhancement to the material of the basefluid and nanoparticles, nanofluid composition (nanoparticle concentration), nanoparticle size, temperature of nanofluid, viscosity (enhancement), type of experimental set up, flow status (i.e. laminar or turbulent), possible gravitational effects (e.g. for convective heat transfer), as well as any other interesting observation (see database tables). Finally, the proposed mechanisms for the observed heat transfer anomalies were identified (the assembled database, which was used for the presented review can be found in Tables 1, 2, 3, 4, 5, 6, 7 and 8).

**Table 1:** Index Number Table

Index Number	Proposed Augmentation Mechanism Theory	Experimental Apparatus
**-**	none mentioned	

**1**	Brownian Motion augmentation theory	Flow in tube or microchannel

**2**	Shear thinning behaviour of flows	transient hot-wire in stationary fluid

**3**	Interfacial layer theory (Kapitza resistance)	Specialised instrument for measuring thermal conductivities/viscosities etc

**4**	Electrical Double Layer (EDL)	Theoretical investigation

**5**	Phonon transfer	Specialised application

**6**	Aggregation and diffusion	Flow over flat heated plates

**7**	Flattening of velocity profile due to viscosity	Quenching

**8**	Thermal conductivity enhancement alone	Heated Wire

**9**	Deposition of nanolayer on heating surface	
	
**10**	Passive/active mode of heat transfer	
	
**11**	Long range structural disjoining pressure	
	
**12**	Near field radiation	
	
**13**	Thermophoresis forces	

**Table 2:** Experiments focusing on heat transfer of Carbon Nanotube - Nanofluids

Paper Reference No	keff/kNF Conduction	keff/kNF Convection/Mixed	NP Material	NP size, (nm unless specified)	BF Material	Φ,(vol% Unless specified)	T test, (K)	Experimental Apparatus Index No	Mechanism Index No	μNF/μBF	Flow Status	EffectsOf Gravity	PBHT	CHT	Notes
[66]	1.20	-	MWNT	10-20nm*1-2 μm	water	2%wt	303	1	1	1	1,2	-	-	-	-

[66]	1.59	-	MWNT	10-20nm*1-2 μm	Water	1%wt	332	1	1	1	1,2	-	-	-	-

[122]	1.07	-	MWNT	15nm*30 μm	DW	1%vol	-	2	-	-	-	-	-	-	-

[122]	1.13	-	MWNT	15nm*30 μm	EG	1%vol	-	2	-	-	-	-	-	-	-

[122]	1.20	-	MWNT	15nm*30 μm	DE	1%vol	-	2	-	-	-	-	-	-	-

[29]	1.18	-	MWNT	-	water	0.1%vol	-	1	2	<1	1,2	-	-	-	-

[29]	1.37	3.50	MWNT	-	water	0.5%vol	-	1	2	<1	1,2	-	-	-	-

**Table 3:** Experiments focusing on Conduction heat transfer

Paper Reference No	keff/Knf Conduction	keff/kNF Convection/Mixed	NP Material L	NP size, (nm unless specified)	BF Material L	Φ,(vol% Unless specified)	T test, (K)	Experiment al Apparatus Index No	Mechanism Index No	μNF/μBF	Flow Status	Effects of Gravity	PBHT	CHT	Notes
[113]	1.35	-	ZnO	77	3:2 mass EG: Water	4.0000	368	3	-	-	-	-	-	-	
		
[113]	1.42	-	ZnO	29		4.0000	368	3	-	-	-	-	-	-	
		
[113]	1.49	-	ZnO	29		7.0000	363	3	-	-	-	-	-	-	
		
[113]	1.60	-	CuO	29		6.0000	363	3	-	-	-	-	-	-	
		
[113]	1.69	-	Al_2_O_3_	53		10.0000	365	3	-	-	-	-	-	-	

[24]	1.07	-	Al_2_O_3_	150	water	1.0000	344	2	1	-	-	-	-	-	

[24]	1.10	-	Al_2_O_3_	11	water	1.0000	344	2	1	-	-	-	-	-	

[24]	1.15	--	Al_2_O_3_	47	water	1.0000	344	2	1	-	-	--	-	-	

[24]	1.29	-	Al_2_O_3_	47	Water	4.0000	344	2	1	-	-	-	-	-	

[73]	1.11	-	Al_2_O_3_	36	water	10.0000	294	2	-	-	-	-	-	-	not large differences generally found in this experiment with varying T, Φ and material
	
[73]	1.12	-	Al_2_O_3_	47	water	10.0000	294	2	-	-	-	-	-	-	

[73]	1.11	-	CuO	29	water	10.0000	294	2	-	-	-	-	-	-	average temperature used (very narrow T range) hence very narrow change in results found (average will be used again) Note LARGE viscosity increase with ΔT around 10K
	
[33]	1.05	-	TiO_2_	21	water	2.0000	294	2	-	+5-15%	-	-	-	-	
	
[118]	1.24	-	Cu_2_O		water	-	294	2	-	-	-	-	-	-	

[59]	-	-	-	-	-	-	-	-	1	-	-	-	-	-	theoretical investigation

[62]	1.11	-	Al_2_O_3_	150	water	1.0000	334	2	3	-	-	-	-	-	averaged values used
	
[62]	1.12	-	Al_2_O_3_	80	EG	1.0000	334	2	3	-	-	-	-	-	
	
[62]	1.12	-	Al_2_O_3_	80	water	1.0000	334	2	3	1.82	-	-	-	-	
	
[62]	1.18	-	TiO_2_	15	EG	5.0000	334	2	3	-	-	-	-	-	
	
[62]	1.37	-	Al	80	Engine Oil	3.0000	334	2	3	-	-	-	-	-	
	
[62]	1.45	-	Al	80	EG	5.0000	334	2	3	-	-	-	-	-	
	
[62]	2.60	-	CNT	0	Engine Oil	1.0000	334	2	3	-	-	-	-	-	
	
[62]	-	-	TiO_2_	15	Water		334	2	3	1.85	-	-	-	-	

[31]	>1	-	-	-	-	-	-	-	-	-	-	-	-	-	theoretical investigation

[48]	1.08	-	Au	17	Water	0.0003	335	4	1,4	-	-	-	-	-	-

[48]	1.10	-	Al_2_O_3_	150	water	4.0000	344	4	1,4	-	-	-	-	-	-

[48]	1.12	-	Al_2_O_3_	47	water	1.0000	344	4	1,4	-	-	-	-	-	-

[42]	1.14	-	Cu	10	EG	0.5500	-	-	3	-	-	-	-	-	-

[42]	1.18	-	Fe	10	EG	0.5500	-	-	3	-	-	-	-	-	-

[34]	1.15	-	Al_2_O_3_	35	EG	5.000	-	-	-	-	-	-	-	-	

[34]	1.20	-	CuO	35	EG	4.0000	-	-	-	-	-	-	-	-	

[34]	1.40	-	Cu	10	EG	0.3000	-	-	-	-	-	-	-	-	

[21]	>1	-	CuO	80*20	Water	0.4000	-	1	-	>1 small	1,2	-	-	-	Turbulent and laminar flow must be present (see pressure diagrams - kick after a point indication of flow turning into turbulent with increased pressure losses). Furthermore, increase in performance observed under specific conditions (e.g. Low flow rates and high temperatures)

[63]	1.05	-	Al_2_O_3_	150	water	5.0000	-	-	3	-	-	-	-	-	-

[63]	1.24	-	Al_2_O_3_	80	water	5.0000	-	-	3	-	-	-	-	-	theoretical investigation

[76]	1.12	-	Al_2_O_3_	38	water	5.0000	-	-	3	-	-	-	-	-	layering theory investigated and found inadequate to account for the results obtained

[64]	>1	-	CuO	28.6	water	4.0000	-	-	1	>1	-	-	-	-	theoretical investigation

[71]	1.07	-	SiO_2_	9	water	14.6000	294	2	-	-	-	-	-	-	Very high concentrations used up to 30%. Used the lowest ones investigated to have a more concise records for comparison with the other papers reviewed. Moreover paper supports that there is no solid indication of anomalous increase in the thermal conductivities of NF

[15]	1.15	-	Al_2_O_3_	38.4	water	1.0000	320	-	1,3,5	-	-	-	-	-	-

[15]	1.22	-	Al_2_O_3_	38.4	water	4.0000	320	-	1,3,5	-	-	-	-	-	theoretical investigation
	
[15]	1.35	-	Cu	10	EG	2.0000	303	-	1,3,5	-	-	-	-	-	
	
[15]	1.20	-	CuO	15	EG	5.0000	-	-	3	-	-	-	-	-	
	
[15]	1.80	-	Cu	3	EG	5.0000	-	-	3	-	-	-	-	-	
	
[9]	2.50	-	CNT	2*54	OIL	1.0000	-	-	3	-	-	-	-	-	

[39]	1.23	-	Al_2_O_3_	35	water	5.0000	-	-	3	-	-	-	-	-	-

[39]	1.25	-	CuO	35	water	4.2000	-	-	3	-	-	-	-	-	-

[39]	1.30	-	Al_2_O_3_	35	EG	6.0000	-	-	3	-	-	-	-	-	average value used

[50]	1.30	-	Al	90	water	5.0000	324	3	1,6	-	-	-	-	-	-

[90]	1.03	-	Au Citrate	15.0000	Toluene	0.001	304	-	-	-	-	-	-	-	Surface Coating
	
[90]	1.05	-	Au Thiolate	3.5000	Toluene	0.0050	334	-	-	-	-	-	-	-	
	
[90]	1.05	-	Au Citrate	15.0000	toluene	0.0003	304	-	-	-	-	-	-	-	
	
[90]	1.07	-	Au Thiolate	3.5000	Toluene	0.0110	304	-	-	-	-	-	-	-	
	
[90]	1.08	-	Au Citrate	15.0000	toluene	0.0003	304	-	-	-	-	-	-	-	
	
[90]	1.09	-	Au Thiolate	Toluene	0.0110	334	-	-	-	-	-	-	-		

[123]	>1	-	-	-	-	-	-	-	1,3	-	-	-	-	-	theoretical investigation - small size, large Φ, large enhancement
	
[94]	>1	-	-	-	-	-	-	-	1	-	-	-	-	-	

[92]	>1	-	-	-	-	-	-	-	1	-	-	-	-	-	theoretical investigation - Brownian dynamic simulation - small size, large Φ large enhancement

[109]	1.05	-	Al_2_O_3_	50	water	2.0	298	-	-	-	-	-	-	-	suspected aggregation at lower NP sizes in this experimental work performed, that's why the conductivity increase for increasing NP size. Authors explain this by implying that the decrease in the NP size leads to increased phonon scattering - decreased NP conductivity
	
[109]	1.06	-	Al_2_O_3_	50	water	3.0	298	-	-	-	-	-	-	-	
	
[109]	1.06	-	Al_2_O_3_	250	water	2.0	298	-	-	-	-	-	-	-	
	
[109]	1.08	-	Al_2_O_3_	50	water	4.0	298	-	-	-	-	-	-	-	
	
[109]	1.09	-	Al_2_O_3_	50	EG	2.0	298	-	-	-	-	-	-	-	
	
[109]	1.09	-	Al_2_O_3_	250	EG	2.0	298	-	-	-	-	-	-	-	
	
[109]	1.09	-	Al_2_O_3_	250	EG	3.0	298	-	-	-	-	-	-	-	
	
[109]	1.11	-	Al_2_O_3_	50	water	3.0	298	-	-	-	-	-	-	-	
	
[109]	1.14	-	Al_2_O_3_	250	EG	3.0	298	-	-	-	-	-	-	-	
	
[109]	1.15	-	Al_2_O_3_	250	Water	3.0	298	-	-	-	-	-	-	-	

[61]	1.02	-	Al_2_O_3_	45	EG	1.0	295	-	-	-	-	-	-	-	3ω method used
	
[61]	1.03	-	Al_2_O_3_	45	EG	2.0	295	-	-	-	-	-	-	-	
	
[61]	1.04	-	Al_2_O_3_	45	water	1.0	295	-	-	-	-	-	-	-	
	
[61]	1.08	-	Al_2_O_3_	45	EG	3.0	295	-	-	-	-	-	-	-	
	
[61]	1.08	-	Al_2_O_3_	45	water	2.0	295	-	-	-	-	-	-	-	
	
[61]	1.10	-	Al_2_O_3_	45	EG	4.0	295	-	-	-	-	-	-	-	
	
[61]	1.11	-	Al_2_O_3_	45	water	3.0	295	-	-	-	-	-	-	-	
	
[61]	1.13	-	Al_2_O_3_	45	water	4.0	295	-	-	-	-	-	-	-	

[91]	>1	-	-	-	-	-	-	-	1	-	-	-	-	-	theoretical investigation

[38]	1.1	-	Ag	60	water	0.3	424	2	1,13	1.1	1	-	-	-	-

[38]	1.15	-	Ag	60	water	0.6	424	2	1,13	1.4	1	-	-	-	-

[38]	1.25	-	Ag	60	water	0.9	424	2	1,13	1.6	1	-	-	-	-

[38]	1.40	-	Ag	60	water	0.3	464	2	1,13	1.5	1	-	-	-	-

[38]	1.80	-	Ag	60	water	0.6	464	2	1,13	1.9	1	-	-	-	-

[38]	2.30	-	Ag	60	water	0.9	464	2	1,13	2.2	1	-	-	-	-

**Table 4:** Experiments focusing on Convection heat transfer

Paper Reference No	keff/kNF Conduction	keff/kNF Convection/mixed	NP material	NP size, (nm unless specified)	BF material	Φ,(vol% unless specified)	T test, (K)	Experimental Apparatus Index No	Mechanism Index No	μ_NF_/μBF	Flow Status	Effects of Gravity	PBHT	CHT	Notes
[43]	-		Al_2_O_3_	-	engine oil	4.4wt	-	5	-	-	-	-	-	-	4WD rotary blade coupling
			

[43]	-	>1	CuO	-		4.4 wt	-	5	-	-	-	-	-	-	
			
[81]	1.03	-	CuO	-	60:40 EG/water	1.0	293	1	-	1.14	-	-	-	-	theoretical investigation
			
[81]	1.06	-	CuO	29		2.0	293	1	-	1.27	-	-	-	-	
			
[81]	1.09	-	CuO	29		3.0	293	1	-	1.69	-	-	-	-	
			
[81]	1.09	1.18	SiO_2_	50		6.0	293	1	-	1.33	-	-	-	-	
			
[81]	1.09	-	SiO_2_	20		6.0	293	1	-	1.41	-	-	-	-	
			
[81]	1.09	-	SiO_2_	100		6.0	293	1	-	1.21	-	-	-	-	
			
[81]	1.12	-	CuO	29		4.0	293	1	-	2.12	-	-	-	-	
			
[81]	1.15	-	CuO	29		5.0	293	1	-	2.60	-	-	-	-	
			
[81]	1.21	1.75	CuO	29		6.0	293	1	-	3.49	-	-	-	-	
			
[81]	1.22	1.36	Al_2_O_3_	53		6.0	293	1	-	1.80	-	-	-	-	

[75]	-	>1	Al_2_O_3_	varying	water	4.0	-	1	-	-	-	-	-	-	theoretical investigation - 2 phase approach showed the smaller the diameter the greater the HTC

[12]	-	1.15	Al_2_O_3_	<100	water	4.0	314	1	6	0.00	-	-	-	-	theoretical investigation - 1 phase approach

[32]	-	-	TiO_2_	21	water	0.2	-	1	-	-	2	-	-	-	negligible HT conduction increase

[60]	-	>1	Al_2_O_3_	45	50:50 EG/water	-	-	2,3	-	<1	-	-	-	-	-

[84]	-	>1	Al_2_O_3_	36	water	2.8	-	5	-	-	2	-	-	-	jet impingement experiment

[17]	-	>1	Cu	42	water	1.0	-	-	-	-	2	-	-	-	theoretical investigation - 2 phase model

[41]	-	1.12	Al_2_O_3_	20	water	0.2	-	1	1,6	-	1	-	-	-	values recorded here for an averaged Pecklet number
	
[41]	-	1.13	Al_2_O_3_	20	water	0.5	-	1	1,6	-	1	-	-	-	
	
[41]	-	1.15	Al_2_O_3_	20	water	1.0	-	1	1,6	-	1	-	-	-	
	
[41]	-	1.22	Al_2_O_3_	20	water	1.5	-	1	1,6	-	1	-	-	-	
	
[41]	-	1.30	Al_2_O_3_	20	water	2.0	-	1	1,6	-	1	-	-	-	
	
[41]	-	1.35	Al_2_O_3_	20	water	2.5	-	1	1,6	-	1	-	-	-	

[18]	1.15	-	Al_2_O_3_	-	water	5.0	-	1	-	-	1	-	-	-	geometry dependent augmentation/deterioration
	
[18]	1.156342	geometry dependent	Al_2_O_3_	-	HFE 7100	5	-	1	-	-	1	-	-	-	

[99]	-	1.03	ZrO_2_	50	water	1.32	-	1	-	-	1	-	-	-	-

[99]	-	1.27	Al_2_O_3_	50	water	6	-	1	-	7.2	1	-	-	-	-

[106]	-	1.08	Al_2_O_3_	30	water	0.3	-	1	1,7	-	1	-	-	-	-

[19]	-	>1	Al_2_O_3_	-	HFC134a	0.1%wt	-	5	-	<1	-	-	-	-	MO: mineral oil used for lubrication inside HFC134a refrigerant fluid along with NPs.Conventionally Polyol- ester (POE) is used as a lubricant
		
[19]	-	>1	TiO_2_	-		0.1%wt	-	5	-	<1	-	-	-	-	MO: mineral oil used for lubrication inside HFC134a refrigerant fluid along with NPs.Conventionally Polyol-ester (POE) is used as a lubricant. Same effect when using the same size Al_2_O_3 _NP

[13]	-	>1	Al_2_O_3_	-	water	0.1	-	5	-	-	-	-	-	-	theoretical investigation - 2 phase approach, smaller diameter, better effects, larger skin friction

[14]	1.04	1.11	Al_2_O_3_	150	water	4%wt	-	1	-	-	1	-	-	-	fully developed region values used here
	
[14]	1.06	1.25	Al_2_O_3_	45	water	4%wt	-	1	-	-	1	-	-	-	

[74]	-	>1	Al_2_O_3_	10	water	2	-	1	1	1	1	-	-	-	theoretical investigation - 2 phase approach-fully developed region values recorded here
[74]	-	>1	Al_2_O_3_	10	water	4	-	1	1	1	1	-	-	-	
	
[74]	-	>1	Al_2_O_3_	10	water	7	-	1	1	1	1	-	-	-	
	
[20]	-	1.12	Al_2_O_3_	100	water	1	-	1	1,6	1.419	1	-	-	-	
	
[20]	-	1.187	Al_2_O_3_	100	water	4	-	1	1,6	1.92	1	-	-	-	

[47]	-	1.32	Al_2_O_3_	170	water	1.8	300	1	-	1	1	-	-	-	average values used

[40]	-	>1	TiO_2_	95	water	0.6	300	1	8	-	1	-	-	-	theoretical investigation 1phase and Langrange & Euler methods used
	
[40]	-	>1	TiO_2_	145	water	0.6	300	1	8	-	1	-	-	-	
	
[40]	-	>1	TiO_2_	210	water	0.6	300	1	8	-	1	-	-	-	

[10]	-	1.3	Cu	-	water	10	-	5	-	-	-	-	-	-	theoretical investigation
	
[10]	-	>1	Ag	-	water	-	-	5	-	-	-	-	-	-	
	
[10]	-	>1	Al_2_O_3_	-	water	-	-	5	-	-	-	-	-	-	
	
[10]	-	>1	CuO	-	water	-	-	5	-	-	-	-	-	-	
	
[10]	-	>1	TiO_2_	-	water	-	-	5	-	-	-	-	-	-	

[77]	1.028192	1	Al_2_O_3_	36	water	1	300	1	-	1.025	1,2	-	-	-	No boiling values recorded
	
[77]	1.030973	1	Al_2_O_3_	36	HFE 7100	1	300	1	-	1.025	1,2	-	-	-	
	
[77]	1.058043	1	Al_2_O_3_	36	water	2	300	1	-	1.050	1,2	-	-	-	
	
[77]	1.061947	1	Al_2_O_3_	36	HFE 7100	2	300	1	-	1.050	1,2	-	-	-	
	
[77]	1.087894	1	Al_2_O_3_	36	water	3	300	1	-	1.075	1,2	-	-	-	
	
[77]	1.09292	1	Al_2_O_3_	36	HEF 7100	3	300	1	-	1.075	1,2	-	-	-	
	
[77]	1.119403	1	Al_2_O_3_	36	Water	4	300	1	-	1.100	1,2	-	-	-	
	
[77]	1.125369	1	Al_2_O_3_	36	HFE 7100	4	300	1	-	1.100	1,2	-	-	-	
	
[77]	1.149254	1	Al_2_O_3_	36	water	5	300	1	-	1.124	1,2	-	-	-	
	
[77]	1.125369	1	Al_2_O_3_	36	HFE 7100	4	300	1	-	1.100	1,2	-	-	-	
	
[77]	1.149254	1	Al_2_O_3_	36	water	5	300	1	-	1.124	1,2	-	-	-	
	
[77]	1.157817	1	Al_2_O_3_	36	HFE 7100	5	300	1	-	1.125	1,2	-	-	-	

[95]	1.028333	-	Al_2_O_3_	42	water	1	294	6	-	-	-	-	-	-	theoretical investigation
	
[95]	1.058333	-	Al_2_O_3_	42	Water	2	294	6	-	-	-	-	-		
	
[95]	1.088333	-	Al_2_O_3_	42	water	3	294	6	-	-	-	-	-	-	
	
[95]	1.118333	-	Al_2_O_3_	42	water	4	294	6	-	-	-	-	-	-	
	
[52]	-	<1	Al_2_O_3_	43.5	water	1	-	5	-	-	-	-	-	-	
	
[52]	-	<1	CuO	11.05	water	1	-	5	-	-	-	-	-	-	
	
[52]	-	<1	JS Clay discs	25diax1thick nes	water	1	-	5	-		-	-	-	-	
	
[101]	-	>1	Cu	100	water	-	-	6	-	-	1	-	-	-	

**Table 5:** Experiments focusing on Natural Convection Heat Transfer

Paper Reference No	keff/kNF Conduction	keff/kNF Convection/mixed	NP material	NP size, (nm unless specified)	BF material	Φ,(vol% unless specified)	T test, (K)	Experimental Apparatus Index No	Mechanism Index No	μ_NF_/μBF	Flow Status	Effects of Gravity	PBH T	CHT	Notes
[51]	-	>1	-	-	-	-	-	2	-	-	-	significant	-	-	theoretical investigation
			
[82]	-	>1	Al_2_O_3_	60	water	0.3-2%	-	1	-	1	-		-	-	
			
[87]	-	>1	Al_2_O_3_	-	water	-	-	2	-	-	-		-	-	
			
[87]	-	>1	Cu	-	water	-	-	2	-	-	-		-	-	
			
[87]	-	>1	TiO_3_	-	water	-	-	2	-	-	-		-	-	
			
[110]	-	>1	-	-	-	-	-	5	-	-	-		-	-	
			
[35]	-	>1	Ag	-	water	-	-	5	-	-	-		-	-	
			
[35]	-	>1	Al_2_O_3_	-	water	-	-	5	-	-	-		-	-	
			
[35]	-	>1	Cu	-	water	-	-	5	-	-	-		-	-	
			
[35]	-	>1	CuO	-	water	-	-	5	-	-	-		-	-	
			
[35]	-	>1	TiO_2_	-	water	-	-	5	3	-	-		-	-	
			
[46]	-	>1	Cu	10	water	-	-	2	1,3,6	-	-		-	-	

**Table 6:** Experiments focusing on Pool Boiling and Critical Heat Flux heat transfer

Paper Reference No	keff/kNF Conduction	keff/kNF Convection/mixed	NP material	NP size, (nm unless specified)	BF material	Φ,(vol% unless specified)	T test, (K)	Experimental Apparatus Index No	Mechanism Index No	μNF/μBF	Flow Status	Effects of Gravity	PBHT	CHT	Notes
[69]	-	-	Ag - silver sphere	35	water	0.5%wt	364	7	9	-	-	-	<1	-	initially washed sphere quenched from 974K
			
[69]	-	-		35	water	1%wt	364	7	9	-	-	-	<1	-	
			
[69]	-	-		35	water	2%wt	364	7	9	-	-	-	<1	-	
			
[69]	-	-		35	water	4%wt	364	7	9	-	-	-	<1	-	
			
[69]	-	-		25	water	0.125%wt	364	7	9	-	-	-	>1	-	
			
[69]	-	-		25	water	0.25%wt	364	7	9	-	-	-	>1	-	
			
[69]	-	-		25	water	0.5%wt	364	7	9	-	-	-	>1	-	
			
[69]	-	-		25	water	1%wt	364	7	9	-	-	-	>1	-	

[115]	-	-	Al_2_O_3_	220	Trypan Blue	-	-	5	10	-	-	-	>1	-	-
		
[115]	-	-	Au (Shells)	170		-	-	5	10	-	-	-	>1	-	-
		
[115]	-	-	Au (spheres)	30		-	-	5	10	-	-	-	>1	-	-
		
[115]	-	-	Au (Rods)	14*45		-	-	5	10	-	-	-	>1	-	-

[57]	-	-	Al_2_O_3_	47	water	0.1	-	8	9	-	-	-	-	1.78	unwashed heating surface values used here. Max values used. When CHT>1 then PBHT is inferred to be >1 as well
	
[57]	-	-	SiO_2_	90	water	0.1	-	8	9	-	-	-	-	2.00	
	
[57]	-	-	TiO_2_	85	water	0.1	-	8	9	-	-	-	-	2.75	
	
[57]	-	-	TiO_2_	85	water	1	-	8	9	-	-	-	-	2.70	
	
[56]	-	-	Al_2_O_3_	47	water	0.1	374	8	9	-	-	-	-	1.75	
	
[56]	-	-	TiO_2_	85	water	0.1	374	8	9	-	-	-	-	2.15	

[119]	-	-	-	-	-	-	-	8	11	-	-	-	-	>1	theoretical investigation

[29]	-	-	Al_2_O_3_	30	water	1.25%wt	-	8	9	-	-	-	1.4	-	aggregation is observed with an effective particle size of around 270 nm
	
[108]	-	-	Al_2_O_3_	25	water	2%wt	-	8	6,8	-	-	-	1.3	-	
	
[108]	-	-	SnO_2_	55	water	3%wt	-	8	6,8	-	-	-	1.2	-	

[54]	-	-	Al_2_O_3_	38.8	water	0.1	304	7	9	-	-	-	-	1.50	Stainless Steel Sphere - SS, Zircalloy Sphere - Zry quenched from 1304K
	
[54]	-	-	Al_2_O_3_	38.8	water	0.1	304	7	9	-	-	-	-	2.37	
	
[54]	-	-	Diamond	165.4	water	0.1	304	7	9	-	-	-	-	1.08	
	
[54]	-	-	diamond	165.4	water	0.1	304	7	9	-	-	-	-	0.60	

[54]	-	-	SiO_2_	32.9	water	0.1	304	7	9	-	-	-	-	1.32	SS sphere

[54]	-	-	SiO_2_	32.9	water	0.1	304	7	9	-	-	-	-	1.54	Zry sphere

[112]	-	-	TiO_2_	21	HCF 141b	0.05	-	8	-	-	-	-	<1	-	Heating surface washed after each trial

[125]	-	-	Al_2_O_3_	-	water	0.05 g/l	334	8	-	-	-	-	1	2.00	Heating surface washed after each trial

[3]	-	-	Al_2_O_3_	20	water	1	371	8	9	-	-	-	1.4	-	heavily agglomerated NF. If greatly sub cooled NF used there is degradation of heating wire

[68]	-	-	CuO	30	water	1%wt	-	8	9	-	-	-	1.25	1.50	Atmospheric Pressure

[68]	-	-	CuO	30	water	1%wt	-	8	4,6,9	-	-	-	2.5	3.00	Lowered Pressure

[55]	-	-	Al_2_O_3_	47	water	0.001	-	8	9	-	-	-	-	1.70	Saturated CHT
	
[55]	-	-	Al_2_O_3_	47	water	0.1	-	8	9	-	-	-	-	1.70	
	
[55]	-	-	TiO_2_	23	water	0.1	-	8	9	-	-	-	-	2.00	
	
[72]	-	-	Al_2_O_3_	22.6	water	0.08%wt	374	8	9	-	-	-	-	1.50	
	
[72]	-	-	Al_2_O_3_	46	water	0.08%wt	374	8	9	-	-	-	-	1.45	
	
[72]	-	-	BiO_2_	38	water	0.01%wt	374	8	9	-	-	-	-	1.33	

**Table 7:** Experiments focusing on Rheological Studies

Paper Reference	keff/kNF Conduction	keff/kNF Convection/mixed	NP material	NP size, (nm unless specified)	BF material	Φ,(vol% Unless specified)	T test, (K)	Experimental Apparatus Index No	Mechanis m Index No	μNF/μBF	Flow Status	Effects of Gravity	PBHT	CHT	Notes
[23]	-	1.08	TNT	10X100	EG	1		-	1,2,6	1.35	-	-	-	-	high shear viscosity recorded here
	
[23]	-	1.15	TNT	10X100	EG	1.75		-	1,2,6	1.75	-	-	-	-	

[93]	-	-	Fe_2_O_3 _- PEO dispersant	30	water	3	299	-	2	1.015	-	-	-	-	high shear viscosity recorded here, averaged values
	
[93]	-	-	Fe_2_O_3 _- PVP dispersant	30	water	3	299	-	2	1.07	-	-	-	-	

[83,85]	-	-	Al_2_O_3_	36	water	3	290	3	-	1.3	-	-	-	-	the effect of rising temperature reduces the effective viscosity. However, the values for augmented temperature for viscosity are not recorded here as they are a result of unstable and damaged NF due to the surfactant change of composition
	
[83,85]	-	-	Al_2_O_3_	36	water	6	290	3	-	2	-	-	-	-	
	
[83,85]	-	-	Al_2_O_3_	36	water	10	290	3	-	3.1	-	-	-	-	
	
[83,85]	-	-	Al_2_O_3_	47	water	1	290	3	-	1.4	-	-	-	-	
	
[83,85]	-	-	Al_2_O_3_	47	water	4	290	3	-	3	-	-	-	-	
	
[83,85]	-	-	Al_2_O_3_	47	water	9	290	3	-	5.3	-	-	-	-	
	
[83,85]	-	-	CuO	29	water	1	290	3	-	1.35	-	-	-	-	
	
[83,85]	-	-	CuO	29	water	4	290	3	-	2.5	-	-	-	-	
	
[83,85]	-	-	CuO	29	water	9	290	3	-	4	-	-	-	-	

**Table 8:** Various experiments not falling into the previous categories

Paper Reference No	keff/kNF Convection	keff/kNF Convection/mixed	NP material	NP size, (nm unless specified)	BF material	Φ,(vol% unless specified)	T test, (K)	Experimental Apparatus Index No	Mechanism Index No	μ_NF_/μBF	Flow Status	Effects of Gravity	PBHT	CHT	Notes
[53]	1.4	-	CNC	15	water	4.2 wt%	299	2	-	1.11	-	-	-	-	-

[22]	1.05	-	SiO_2_	10	water	16	-	-	-	-	-	-	-	-	-

[22]	1.08	-	SiO_2_	15	water	16	-	-	-	-	-	-	-	-	-

[22]	1.16	-	SiO_2_	30	water	16	-	-	-	-	-	-	-	-	-

[49]	>1	>1	-	-	-	-	-	-	3,6,12	>1	-	-	-	-	theoretical investigation
	
[121]	-	>1	Al_2_O_3_	42.5	water	-	-	-	1,13	-	-	-	-	-	

[126]	-	1.60	SiC	170	water	3.7	320	1	1,13	>1	2	-	-	-	lower viscosity rather than using Al2O3

[45]	-	1.01	Al_2_O_3_	150	EG	0.5	294	-	1	-	-	-	-	-	theoretical investigation
	
[45]	-	1.03	Al_2_O_3_	150	EG	0.5	300	-	1	-	-	-	-	-	
	
[45]	-	1.03	Al_2_O_3_	150	EG	0.5	309	-	1	-	-	-	-	-	
	
[45]	-	1.05	Al_2_O_3_	150	EG	0.5	324	-	1	-	-	-	-	-	
	
[45]	-	1.06	Al_2_O_3_	150	EG	2	300	-	1	-	-	-	-	-	
	
[45]	-	1.11	Al_2_O_3_	11	EG	1	294	-	1	-	-	-	-	-	
	
[45]	-	1.12	Al_2_O_3_	150	EG	3	300	-	1	-	-	-	-	-	
	
[45]	-	1.13	Al_2_O_3_	11	EG	1	309	-	1	-	-	-	-	-	
	
[45]	-	1.16	Al_2_O_3_	11	EG	1	324	-	1	-	-	-	-	-	
	
[45]	-	1.17	Al_2_O_3_	60	EG	2	300	-	1	-	-	-	-	-	
	
[45]	-	1.35	Al_2_O_3_	60	EG	5	300	-	1	-	-	-	-	-	
	
[58]	-	1.10	Al_2_O_3_	80	water	2	-	-	1	-	-	-	-	-	
	
[58]	-	1.15	Cu	100	water	2	-	-	1	-	-	-	-	-	
	
[58]	-	1.55	Cu	100	water	5	-	-	1	-	-	-	-	-	

[86]	-	>1	Al_2_O_3_	20	water	2	-	5	1,9	-	-	-	>1	-	averaged values used. Thermosiphon experiment

[88]	-	>1	CuO	30	water	4	329	5	-	>1	2	-	-	-	-

[102]	-	-	Al	60	Ethanol	2	310	5	-	>1	-		>1	-	-

[89]	1.039539	>1	CuO	30	water	2	-	5	-	1.3	2	-	-	-	-

[89]	1.059308	>1	Al_2_O_3_	20	water	2.9	-	5	-	2.9	2	-	-	-	-

[89]	1.059308	>1	CuO	40	water	3	-	5	-	-	2	-	-	-	-

[89]	1.059308	>1	TiO_2_	-	water	2.4	-	5	-	2	2	-	-	-	-

[89]	1.067545	>1	Al_2_O_3_	11	water	4	-	5	-	-	2	-	-	-	-

[89]	1.102142	>1	CuO	30	water	4	-	5	-	2	2	-	-	-	-

[89]	1.186161	>1	CuO	30	water	8	-	5	-	5.6	2	-	-	-	-

The methodology for the capturing of the findings (numerical and theoretical) from each publication and ensure repeatability of data collection and analysis is as follows:

a. It was decided to limit the data gathering for volumetric concentrations of nanoparticles (Φ) up to 10% (focus group).

b. Information was presented on diagrams only when adequate number of cases was available in order to be able to approximately describe the shape of the resulting graph.

c. In cases where Dynamic Light Scattering (DLS) or a Brunnauer-Emmet-Teller (BET) sizing method was used in conjunction with a Transfer Electron Microscopy (TEM) or Scanning Electron Microscopy (SEM) method, the latter sizing values were preferred over the former ones as they provide better accuracy (DLS and BET methods both take into account the hydrodynamic size of particles with the assumption of sphericity instead of their actual dimensions. This incurs problems when the nanoparticles are clustered/agglomerated or not spherical).

d. In the cases where the Pool Boiling Heat transfer (PBHT) or Critical Heat Flux (CHF) were considered, values from experiments representing a real and practical engineering application were recorded over the rest.

e. In the rare case where nanoparticle concentrations were represented as mass fraction quantities, a volumetric conversion, according to Equation 1 was used [7].(1)

f. When the mode of heat transfer was not clearly stated or was not evident from the experiment (for example if heat transfer mode was purely via conduction/convection), then the experimental values were sorted into the convection/mixed convection heat transfer class (when both modes are present, it is expected that the effects of convection would prevail over the effects of conduction).

**Figure 1:**
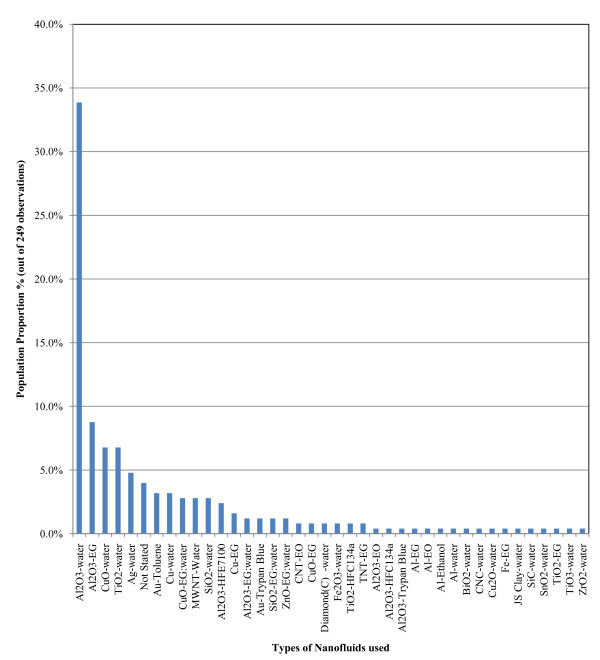
**Nanofluid type distribution**.

Table 9 displays an average price list of different nanoparticle materials, while Table 10 and Figure 1 show the nanofluid types in the literature. It is evident that the cost of particular type(s) of nanoparticles heavily controlled the available study. As a consequence, the statistical results of this paper are heavily inclined towards indicating the thermal performance of Al_2_O_3_-water type nanofluids.

**Table 9:** Most common Nanoparticle materials along with their indicative price ($) per 100 g

Material	Indicative Price ($/100 g)
**Al (Aluminium)**	380
**Al_2_O_3 _(Aluminium Oxide)**	70
**Cu (Copper)**	500
**CuO (Copper Oxide)**	75
**Au (Gold)**	5,500
**Ag (Silver)**	400
**SiO_2 _(Silica Dioxide)**	70
**TiO_2 _(Titanium Oxide)**	80
**Carbon Nanotubes**	930-12,500

**Table 10:** The four most probable Nanofluids found in the literature

Type of Nanofluid Used	Sample Percentage	Number of Corresponding Observations
**Al_2_O_3 _- Water**	33.9	85
**Al_2_O_3 _- Ethylene Glycol (EG)**	8.8	22
**CuO - Water**	6.8	17
**TiO_2 _- Water**	6.8	17
**Total**	56.3	141

## Thermal performance studies

Previous investigators chose to carry out their studies either via the experimental or the analytical route. For the former one, the majority of researchers selected simple experiments (e.g. simple heated pipe/duct flow or stationary flow experiments) using various combinations of nanofluid concentrations and materials under different heat input conditions. The simple experiments provided more insight into the actual physics of heat transfer in nanofluids whilst the more complex experiments usually gave information concerning the practical usage of particular nanofluid compositions and types for certain applications, with little or no referral to the employed theories for heat transfer.

Analytical-computational methods involve the formulation of semi-empirical correlations in order to predict the behaviour of nanofluids. The most common analytical methods are based on the renovated Maxwellian [8], Equation 2, or renovated Hamiltonian-Crosser equation models [9], Equation 3, to be able to predict the effective heat conduction in a nanofluid. Additional components are usually added to the equations to take into account the Brownian motion heat transfer mechanism.(2)(3)

Equations 2 and 3 rely on the molecular layering theory, i.e. the presence of nanolayers with reduced thermal resistance covering the surface of each nanoparticle. The renovated Hamiltonian-Crosser model equation is assumed to be more accurate, as the shape of the solid nanoparticles is taken into account (sphericity), while the renovated Maxwellian model only assumes spherical particles and works well for nanoparticle diameters that are less than 10 nm [8].

For the other heat transfer modes (apart of heat conduction), the formulation of further equations to include additional parameters (e.g. density changes, buoyancy forces, gravitational forces, etc.), has its foundations on Equations 2 and 3.

The critical issue with numerical simulations and semi-empirical correlations is that the majority of researchers predetermined, to some degree, the physical mechanisms underlying behind the anomalous heat transfer characteristics in nanofluids. For example, some semi-empirical correlations are based on fitting experimental measurements determined for specific applications. As a result, with the physical understanding of the heat transfer mode mechanisms yet unknown, it becomes trivial to solemnly rely on such simulations and equations to hold valid for a general range of nanofluid compositions, types and application (e.g. as coolants in various heat exchanger designs).

## Heat transfer characteristics [1-128]

In the following section, the heat transfer characteristics of nanofluids are considered. Information was collected from the literature and processed to reveal the thermal performance of nanofluids for different heat transfer modes (purely conductive, convective/mixed, pool boiling and CHF). Information, regarding the mechanisms that various researchers employed to describe the anomalous heat transfer, was also collected to allow the evaluation of the most statistically occurring patterns for each heat transfer mode.

Finally, a cross-correlation of the findings between the different levels of analysis (explained in "Methodology of statistical analysis" section) was also considered to evaluate the observations and reveal any possible trends linking the thermal performance characteristics of nanofluids with their by part properties (i.e. consistency and application). Furthermore, the focused samples of level 3 of the analysis provided further information about the parameters controlling the thermal performance characteristics of nanofluids.

### General observations: level 1 analysis

Level 1 of the analysis considers the entire sample record collected from the literature. It aims to present a general idea of the thermal performance of nanofluids for different heat transfer modes.

#### Heat transfer characteristics

##### a. Heat transfer enhancement studies purely via conduction (130 observations)

**Figure 2:**
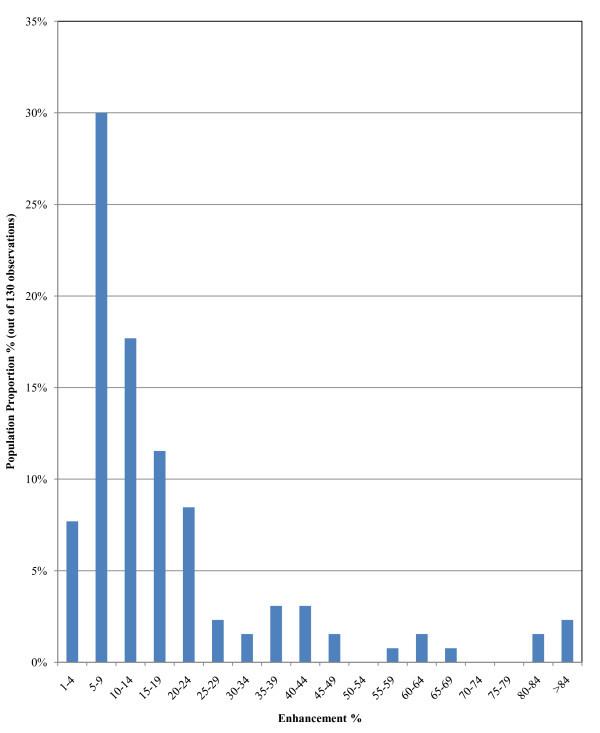
**Probability function of enhancement of heat transfer due to conduction**.

Strong evidence of thermal conductivity enhancement exists, as indicated by the histogram of the findings of Figure 2. An enhancement lying between 5 and 9% was observed for 30% of the sample. The variation around the 5-9% enhancement range is large. However, the majority of the remaining observations are in the 1-4% and 10-24% enhancement ranges, representing around 45% of the sample. The remaining data (around 25% of the sample) indicate enhancement above 29% and some even larger than 84%. Therefore, there is a need for additional understanding of the origin of the resulting enhancement of heat transfer due to conduction.

##### b. Heat transfer enhancement studies via convection/mixed heat transfer mode (91 observations)

**Figure 3:**
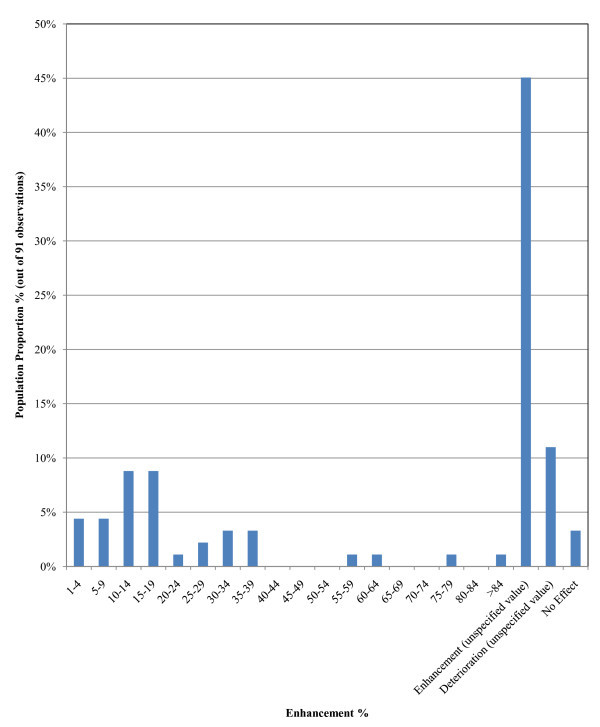
**Probability function of enhancement of heat transfer due to convection/mixed**.

Strong evidence of heat transfer enhancement by nanofluids for convective or mixed heat transfer mode is indicated in the histogram of Figure 3. Most data indicate a convective heat transfer coefficient enhancement between 10 and 19% (18% of the sample). However, the spread of the enhancement results is very large. The majority of the results (around 45% of the sample) indicated unspecified enhancement. There is also weak statistical indication of nanofluids causing deterioration of the heat transfer coefficient (11% of the sample) and an even smaller percentage of the sample indicating no enhancement at all (3% of the sample). Therefore, the statistical analysis for convective heat transfer is less consistent than for conduction, which supports the need for more research.

##### c. PBHT enhancement studies (22 observations)

**Figure 4:**
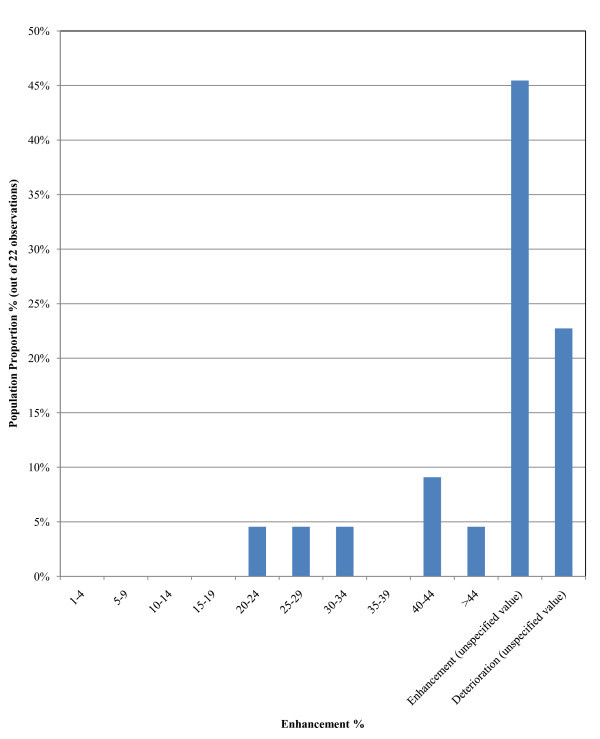
**Probability function of enhancement of heat transfer due to pool boiling**.

Strong evidence of enhancement of heat transfer due to pool boiling is indicated in the histogram of Figure 4. Most data reporting specific values show an improvement of the PBHT coefficient between 40 and 44% (9% of the sample). However, the majority of the results (45% of the sample) indicate an unspecified enhancement, while there is an indication of deterioration with moderate statistical importance (23% of the sample) and a weak statistical percentage of the considered sample indicating no enhancement at all (5% of the sample). However, the number of publications for PBHT is low and, as a consequence, the findings have lower confidence level. In addition, the complexity of the physics of PBHT can cause large variation in the observed enhancement and the lack of understanding does not allow the assessment of optimised operation with PBHT.

##### d. CHF enhancement studies (23 observations)

**Figure 5:**
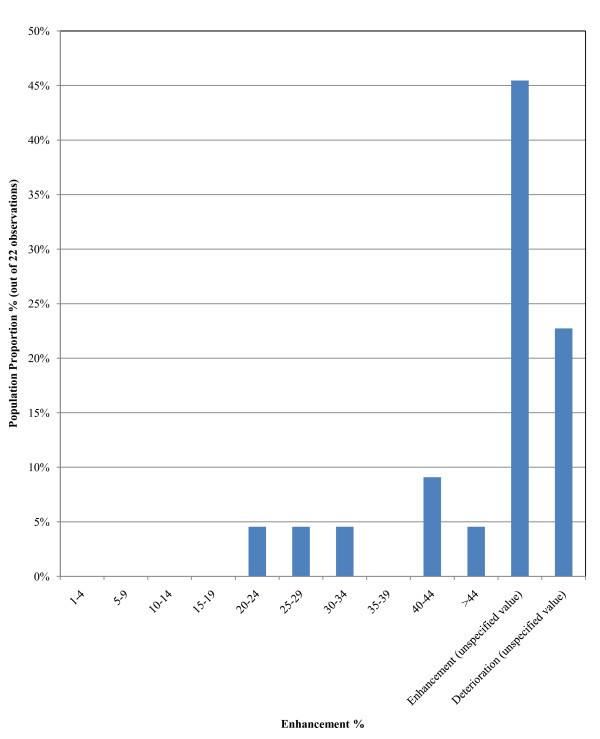
**Probability function of enhancement of heat transfer due to CHF**.

Strong evidence of enhancement of **CHF **in boiling applications is indicated in the histogram of Figure 5. Most observations show an improvement of the CHF coefficient lying between 100 and 200% (35% of the sample). There is a weak statistical percentage of the considered population indicating deterioration (4% of the sample). However, the spread of the results is large and the confidence level of the findings is low. Since several publications have reported very large enhancement of CHT, it is important to understand the origin of CHT enhancement in nanofluids.

##### e. Proposed physical mechanisms for the anomalies of heat transfer

An outline of all the proposed mechanisms for each type of heat transfer study is presented. These mechanisms (or a combination of more than one mechanism) are used by researchers in the literature to explain the augmentation of the heat transfer coefficient in nanofluids. The proposed mechanisms are briefly explained first before the findings of the statistical analysis are presented. The findings are considered jointly for conduction and convection and for pool boiling and CHF.

##### Conduction/convection/mixed mode heat transfer studies (85 observations)

The proposed mechanisms for the enhancement of heat transfer in conduction, convection or for mixed conditions in the literature are described below. Example references of papers containing the explanation of the theory are also provided.

*Brownian motion*

Many researchers believe that there is an apparent enhancement of heat transfer due to Brownian motion of nanoparticles. Their speculations rely on the fact that nanoparticles provide larger surface area for molecular collisions. The higher momentum of nanoparticles (higher mass concentrations compared to the host fluid molecules) are believed to carry and transfer thermal energy more efficiently at greater distances inside the basefluid before they release it in a colder region of the fluid (small packets of energy) [42].

*Interfacial layer theory (Kapitza resistance)*

The Kapitza resistance is a thermal boundary resistance arising from thermal energy carrier scattering at an interface (scattering of phonons and electrons). The type of carrier scattered will depend on the materials governing the interfaces. In liquid-solid interfaces (e.g. nanoparticle-base fluid interfaces), the boundary resistance is believed to decrease hence the overall thermal resistance of the system (e.g. a nanofluid in this case) is believed to reduce [70].

*Aggregation and diffusion*

This mechanism suggests that there is a formation of a linear assembly of nanoparticle chains upon their suspension in the host fluid. The occurrence of this chain assembly is speculated to provide a faster path for heat transfer through the nanofluid (faster heat diffusion) [65].

*Electrical double layer (EDL) theory*

This mechanism proposes an alteration of the strength of intermolecular interaction forces that in effect change the mean free path of the nanoparticles and hence augmenting the heat transfer of molecules [48].

*Flattening of velocity profile due to viscosity*

This mechanism proposes that the viscosity change of nanofluids leads to a more uniform velocity profile for flows in pipes and ducts than the expected parabolic velocity profile (Poiseuille flow). The increased near wall velocity is believed to provide an increase in the convective heat transfer coefficient observed in these applications [106].

*Near field radiation*

Some researchers believe that there is infrared radiation emission and absorption augmentation at the nanoscales (near field radiation). This enhances heat transfer between the heating surface and the nanoparticles, the basefluid molecules and the nanoparticles and between the nanoparticles themselves by a factor of 2-3 compared to the far field radiation estimates [37].

*Thermophoretic forces*

Thermophoretic forces on nanoparticles arise from the presence of temperature gradients in the fluid causing the concentration of nanoparticles to change around heating and cooling sides relative to the mean value. The consequence of this nanoparticle redistribution is the alteration of the heat transfer coefficient accordingly [121].

*Shear thinning behaviour of flows*

Some researchers believe that nanofluids exhibit non-Newtonian characteristics with shear thinning behaviour. The viscosity is believed to reduce at the solid boundaries of a flowing nanofluid, because the shear rate of the nanofluids increases along the walls. This promotes increased heat transfer between the wall and the liquid because the thermal boundary layer width is reduced. It also provides a beneficial lubrication effect [30].

*Phonon transfer*

A few researchers suggested that nanofluids have an increased heat transfer rate due to specialised phonon and electron interaction and scattering at the nanoscales (ballistic heat transport) [64].

*Thermal conductivity enhancement alone*

Some researchers have accounted for the increase of the thermal conductivity alone (without providing more information) to account for the observed enhancement of heat transfer [40].

**Figure 6:**
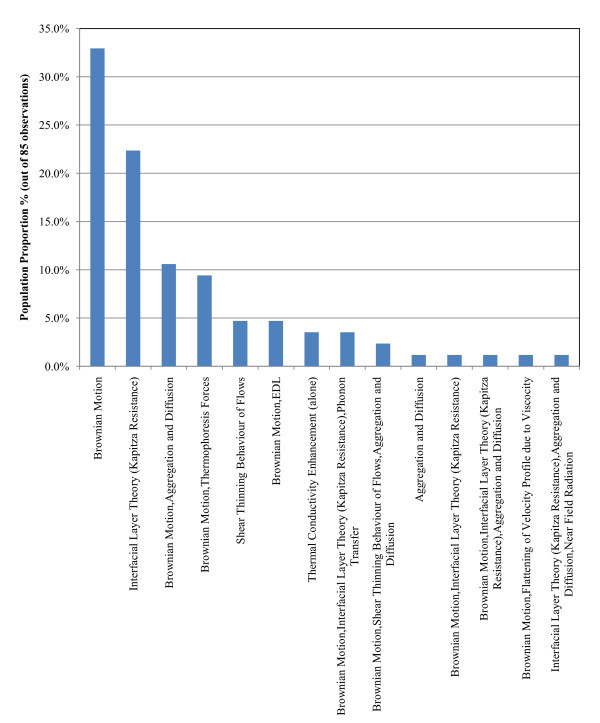
**Probability function of proposed mechanisms to explain anomalous heat transfer (conduction/convection/mixed mode heat transfer studies)**.

Figure 6 presents the histogram of the proposed mechanisms to explain the anomalous heat transfer for conduction, convection and mixed cases in the literature. The observations from Figure 6 are summarised below and there are three most commonly proposed mechanisms:

a. Brownian motion (33% of the sample)

b. Interfacial layer theory (Kapitza resistance) (22.4% of the sample)

c. A combination of the Brownian motion and the aggregation and diffusion theories (11% of the sample).

##### PBHT and CHF enhancement studies (40 observations)

The proposed mechanisms for the enhancement of PBHT and CHF in the literature are described below.

*Deposition of nanoparticles on heating surface*

The vast majority of researchers assume that, for this heat transfer mode, the use of nanofluids leads to a modification of the heating surface. The alteration promotes higher frequency of bubble departure with smaller bubble size. At the same time, there is an increased wettability that inhibits the dry patch development on the heating element, leading to increased CHF [57].

*Passive/active mode of heat transfer*

The passive mode mechanism suggests that nanoparticles provide additional nucleation sides for vapour bubble formation and boiling. The active mode mechanism suggests that nanoparticles provide appropriate surface area for converting infrared Radiation into heat. These two modes are suspected to increase the overall heat transfer coefficient of nanofluids [115].

*Long range structural disjoining pressure*

Confinement of nanoparticles in the meniscus area, supplying liquid to the formation of the vapour bubble at the dry patch, is believed to promote an increased wettability and inhibition of the dry patch development [119]. This leads to increased CHF.

*Electrical double layer (EDL) theory*

This mechanism was also proposed to explain conduction/convection heat transfer enhancement. It is based on a change of the strength of intermolecular interaction forces that modifies the mean free path of the nanoparticles [48].

*Thermal conductivity enhancement alone*

This mechanism was also proposed to explain conduction/convection heat transfer enhancement. It makes use of the increase of the thermal conductivity alone (without providing more information) to account for the observed enhancement of heat transfer [40].

**Figure 7:**
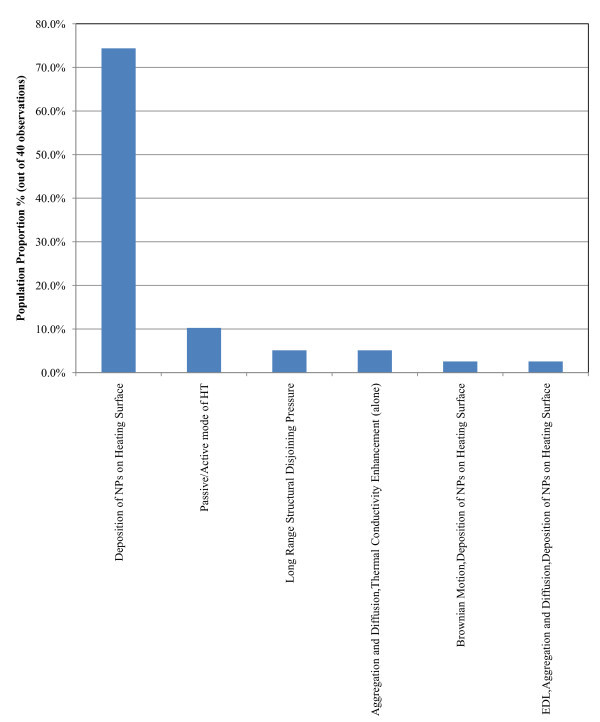
**Probability function of proposed mechanisms to explain anomalous heat transfer (pool boiling heat transfer and CHF heat transfer studies)**.

Figure 7 presents the histogram of the proposed mechanisms to explain the anomalous heat transfer for pool boiling and CHF in the literature. The observations from Figure 7 are summarised below and there are two most commonly proposed mechanisms:

a. Alteration of the heating surface due to the deposition of nanoparticles (75% of the sample)

b. Passive/active heat transfer mode theory (10% of the sample)

In summary, a general overview of the thermal performance for each heat transfer mode was presented. It is evident that the vast majority of publications in the literature indicated that nanoparticles are found to augment the heat transfer coefficient of a given basefluid for every mode of heat transfer.

The most popular mechanisms for explaining the anomalous heat transfer were also presented. All of the proposed mechanisms have not been verified experimentally and as a result these proposals still remain notions of what is theoretically employed by researchers to explain the phenomena.

### Evaluation of trends of specific nanofluids: level 2 analysis

Level 2 of the statistical analysis contains a narrowed down sample of publications. The criterion for selecting the publications of the secondary group of level 2 was the nanofluid material composition. It was decided to select the nanofluid material consistencies that were most commonly used in the literature. This enables the in-depth comparison between observations recorded from different research groups found in the literature, hence allowing the definition of possible trends linking the thermal performance characteristics of nanofluids with their by part properties (such as consistency, temperature of nanofluid, etc). The formation of the secondary group also provides correlation information between the two analysis levels (namely levels 1 and 2) that assists the evaluation of the statistical analysis findings.

#### Nanofluid types considered (249 observations)

A histogram of nanofluid types employed in the literature was presented in Figure 1 and was considered again here to discover which types have been studied most and, hence, allow the creation of secondary focus groups. The selected sample was narrowed to the following nanofluids: Al_2_O_3_-water, Al_2_O_3_-ethylene glycol (EG), CuO-water and TiO_3_-water (see Table 10). The processing of the above level 2 analysis sample indicated that the number of publications for the latter two types of nanofluids was too small to obtain conclusions with reasonable statistical significance. Hence, it was decided to consider only the results for the former two nanofluids (i.e. the Al_2_O_3_-water and Al_2_O_3_-EG).

#### Heat transfer characteristics

The statistical analysis 2 of the thermal performance was performed for each heat transfer mode, when the sample was large enough (above 10 observations) to justify the statistical findings. Histograms of this analysis are not presented here, but the findings are summarised below.

##### a. Heat transfer enhancement via conduction

*Al_2_O_3_-water nanofluids (41 observations)*

Strong evidence of thermal conductivity enhancement is present. Heat transfer enhancement was observed mainly between 5 and 9% (34% of the sample). The variation around the 5-9% enhancement regime was small with the majority of the remaining observations in the enhancement range of 10-14% (32% of the sample).

*Al_2_O_3_-EG nanofluids (11 observations)*

Strong evidence of thermal conductivity enhancement is present. Heat transfer enhancement lying between 5 and 9% was similarly observed (36% of the sample). The variation around the 5-9% enhancement range was again small with the majority of the remaining observations in the 10-14% range (27% of the sample).

The findings for the two nanofluids are complimentary and in agreement with the findings for all types of nanofluids as obtained from the analysis of level 1 and presented in Figure 2.

##### b. Heat transfer enhancement studies via convection/mixed heat transfer mode (91 observations)

*Al_2_O_3_-water nanofluids (36 observations)*

There is strong evidence of heat transfer enhancement with most publications indicating an unspecified value of enhancement (39% of the sample).

*Al_2_O_3_-EG nanofluids (11 observations)*

Strong evidence of heat transfer enhancement is present. Most observations indicate an enhancement between 1-4% and 10-14% (27% of the sample for each range). The spread is small with all results indicating an enhancement around the 1-19% enhancement range.

It should be noted that the findings for the two nanofluids is in agreement with the findings of analysis level 1, as presented in Figure 3.

#### Proposed physical mechanism for anomalous heat transfer

##### a. Conduction/convection/mixed mode heat transfer studies

*Al_2_O_3_-water nanofluids (29 observations)*

a. Brownian motion (28% of the sample)

b. Brownian motion combined with the aggregation and diffusion theory (28% of the sample)

c. Interfacial layer theory (Kapitza resistance) (17% of the sample)

*Al_2_O_3_-EG nanofluids (13 observations)*

a. Brownian motion (85% of the sample)

b. Interfacial layer theory (Kapitza resistance) (15% of the sample).

The most popular proposed mechanism is the Brownian motion of the nanoparticles. This is in agreement with the findings of level 1 analysis presented in Figure 6. Some differences exist for the second and third most popular mechanisms, but both are the same as for the level 1 analysis.

##### b. PBHT and CHF (12 observations)

The sample for the Al_2_O_3_-EG nanofluids was too small to give a credible statistical result. Hence, only the Al_2_O_3_-water nanofluid sample is presented. The most popular proposed mechanisms are:

a. Alteration of the heating surface due to deposition of nanoparticles (83.3% of the sample)

b. Brownian motion combined with the alteration of the heating surface due to deposition of nanoparticles (8.33% of the sample)

c. Deposition and aggregation theory combined with the diffusion and thermal conductivity (alone) (8.33% of the sample).

The alteration of the heating surface by the deposition of nanoparticles is the most popular proposed mechanism for the explanation of enhanced PBHT and CHF. This is in agreement with the findings of Level 1 analysis presented in Figure 7.

##### Scatter diagrams based on level 2 analysis: indication of trends

**Figure 8:**
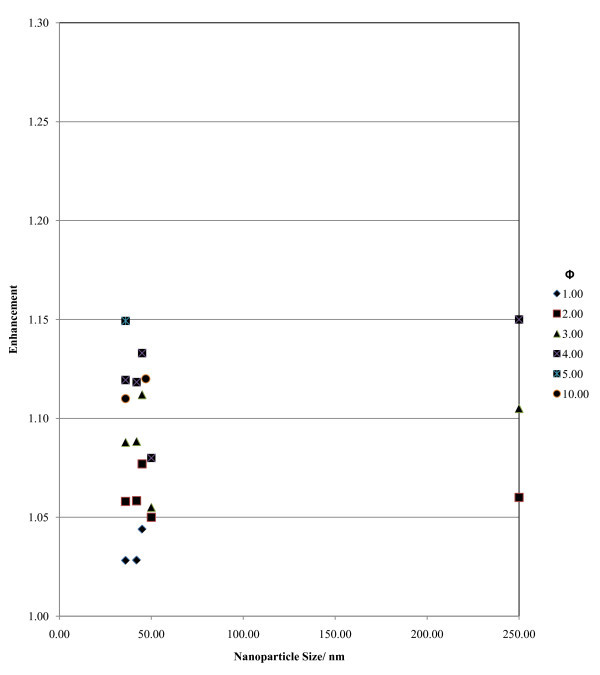
**Sample of one of the scatter diagrams used to extract the trends**. The diagram depicts various results of conductive heat transfer enhancement for the Al_2_O_3_-water type nanofluid at various concentrations (Φ) and at a temperature range of 290-310 K.

**Figure 9:**
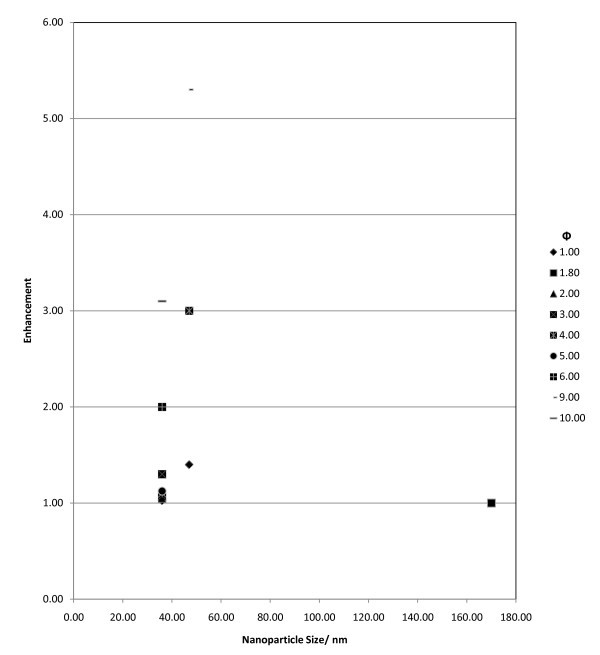
**Sample of one of the scatter diagrams used to extract the trends**. The diagram depicts various results of viscosity enhancement for the Al_2_O_3_-water type nanofluid at various concentrations (Φ) and at a temperature range of 290-310 K.

Level 2 analysis allowed the formation of various scatter diagrams and two of the most representative diagrams are selected and can be seen in Figures 8 and 9. Figure 8 presents the effect of nanoparticle concentration and size on conducting heat transfer for Al_2_O_3_-water nanofluids. Figure 9 shows the effect of nanoparticle concentration and size on the viscosity of the mixture for Al_2_O_3_-water nanofluids. The scatter diagram analysis provided vital information on the links between nanofluids parameters and their thermal performance. The following trends were derived:

a. The level of enhancement for the purely conductive case indicated an increasing trend with increasing nanofluid temperature and nanoparticle concentration, while there is a slight hint of the enhancement increasing with nanoparticle size (see Figure 8).

b. The effective viscosity of the mixture is enhanced and the findings show an increasing trend with decreasing fluid temperature, increasing volumetric concentration. There is also a slight hint of an effective viscosity increase with decreasing nanoparticle size (see Figure 9).

c. The level of enhancement for the convection/mixed heat transfer mode indicated an increasing trend with increasing temperature, volumetric concentration and decreasing nanoparticle size.

Unfortunately, this trend can only be deduced by taking account the entire sample of scatter diagrams produced hence there is not a single representative diagram to display supporting it (contrary to the conductive and viscosity enhancement trends considered).

The trends appear to hold true up to the level where the nanofluid defining qualities (regarding particle suspension and chemical consistency properties as listed in Characteristics of nanofluids section) are still satisfied and the nanoparticle concentrations remain in between the boundaries set in the methodology of observation collection (0.0001-10 vol.%). It should be noted that the observed trends cannot be quantified to evaluate the contribution of each by part property of the nanofluids. The observed trends will be discussed in the next subsection.

Unfortunately, the sample size was not large enough to extract trends regarding PBHT and CHF. Moreover, due to sample size limitation it was also impossible to determine nanoparticle material effects on thermal performance characteristics.

##### Discussion of findings from level 2 analysis

The thermal performance assessment study of the second level agrees to a large degree with the findings of the first level. The cross correlation between the two levels ensures that the narrowed sample still falls into the reliability limits of the study.

The heat transfer enhancement studies for the Al_2_O_3_-water/EG generally indicated strong enhancement for the conduction and convection/mixed modes. More specifically, for the conduction heat transfer enhancement, the two nanofluids performed the same. For the convection/mixed heat transfer mode, the results for the two nanofluids do not correlate to the same level of enhancement.

For the statistically most popular proposed mechanism to explain the anomalous heat transfer. Both types of nanofluids agree on the Brownian motion of nanoparticles being most popular mechanism to explain the phenomena while small deviations appear regarding the secondary and third most popular mechanisms.

In the light of this evidence, it can be concluded that the narrowing of the sample to perform the second level of the analysis can still be accounted creditable, as it generally agrees with the findings from the overall sample of level 1. The findings regarding the thermal performance indicate that the basefluid material has little influence on the heat transfer enhancement in the conduction mode. On the contrary, the basefluid material seems to affect the performance for the convection/mixed heat transfer mode as discrepancies on the performance studies for the two materials were observed.

The observed trends on the effect of nanofluid properties to heat transfer of the trend formation can be accounted to the following for each subsection:

##### Trends for conduction enhancement

The findings indicate that conduction enhancement increases with increasing particle size, increasing nanoparticle concentration and mixture temperature. For this "less active" (compared to the convection/mixed) mode, Brownian motion becomes less pronounced; it appears that this trend is reasonable by referring partially to the second most popular mechanism found in the literature, namely aggregation and diffusion. By increasing the nanoparticle size and concentration, the highly conductive nanoparticles can diffuse the heat faster into the liquid as the thermal energy coefficient for solids is much larger than that of liquids. The increase of particle size provides longer and more effective ground for heat diffusion through each nanoparticle, while the increase of concentration increases the volume of the highly conductive solid available for heat transfer in the nanofluid. The enhancement increases with increasing temperature. This is accounted for by conventional heat transfer mechanisms. The increased temperature leads to statistically more energetic molecules. The statistical thermo mechanics for liquids and solids dictates that the intermolecular interactions will increase (collisions become more frequent and the energy involved per collision increases due to the average molecular speed augmentation). As a consequence, the heat transfer due to conduction is enhanced with increasing temperature. It should be noted that there must be a critical value of nanoparticle size and concentration beyond which the observed trend will reverse. However, there is no information available that will be able to demonstrate this assessment.

##### Trends for convection/mixed enhancement

The convection/mixed heat tramsfer mode is more "energetic" than conduction. Hence, Brownian motion of nanoparticles is more pronounced. The liquid molecules are allowed to move under the influence of buoyant forces arising from density variations inside the liquid. The heat transfer enhancement follows an increasing trend with decreasing nanoparticle size, increasing nanoparticle concentration and increasing temperature. For the latter, the same principle holds true as for conduction enhancement. For the former, at a given volumetric concentration of nanoparticles, the decrease in nanoparticle size results in an increase in the particle surface area available for collisions and at the same time an increase in the number of nanoparticles and a decrease in the corresponding mass per particle in a given volume of a nanofluid (the mass reduction per particle is much smaller compared to the gain of free surface area available for collisions). The increased surface area and number of nanoparticles results into an increased number of collisions between the basefluid molecules and the nanoparticles as well as between the nanoparticles themselves. Moreover, the decreased particle mass and increased collision count and hence overall collision energy involved leads to an increase of the mean free path (according to the most prevailing mechanism found in the literature) and energy content per nanoparticle. Brownian motion is hence augmented giving rise to large local density variations (and hence large buoyancy force variations) that, in turn, provide an enhancement in the convective/mixed heat transfer coefficient.

##### Trends for effective viscosity of nanofluid mixture

The effective viscosity follows an increasing trend with decreasing nanoparticle size, increasing nanoparticle concentration and decreasing temperature. For the latter, the decrease of temperature results in less energetic i.e. more sluggish liquid molecules. The decreasing temperature results in a decreasing kinetic energy of each molecule. The attractive/repulsive intermolecular forces become more pronounced giving rise to an enhancement in viscosity. Additionally, the decrease in particle size for a given volumetric concentration and volumetric amount of a nanofluid results in larger nanoparticle surface area and number count, which consequently results in a rise in the shear stress observed between the solid-solid and solid-liquid interfaces inside the fluid-contrary to the shear thinning mechanism accounted by some researchers. This effect is also augmented by increasing the volumetric concentration of nanoparticles, since the overall nanoparticle surface area and number count of nanoparticles are increased as well.

### Focus on simple experiments: level 3 analysis

It was decided to investigate further the available information by considering publications, which reported simple and well-documented experiments. The simplicity of the experiments will allow focusing on the effects of nanoparticles, while other parameters introduced by the complexity (e.g. geometry) of the experiment will be eliminated. It is expected that these publications will enable a more generic view of the anomalous heat transfer characteristics of nanofluids, while it will also allow other researchers to reconstruct experiments in order to carry out further investigations on the notions and suggestions of previous studies.

It was decided to focus on publications that:

a. include a physical experiment (i.e. eliminate those with computer simulations)

b. consider simple experiments, where concise documentation is available, consisting of:

▪ heat exchange via flow through a duct/pipe (simple pipe flow)

▪ heat exchange in stationary flow, i.e. transient hot-wire experiments

It was hence possible to construct new limited data sets and produce new histograms that enable the extraction of more targeted quantities. Results are presented where the sample size was sufficient to have a statistical importance (at least 10 observations).

#### Transient hot-wire experiments

The transient hot-wire experiments involve conventional conductivity measurements in a stationary fluid by means of the transient hot-wire apparatus and method. This experimental procedure is considered to be one of the most accurate and simple methods used to deduce the thermal conductivity of nanofluids. The experiments are believed to provide an insight in the performance criteria without unnecessary experimental complexities that might affect the results acquired. Heat transfer studies and analysis to determine the proposed mechanisms for heat transfer were performed similarly to level 1 and level 2 of this investigation. No histograms are presented; however, the numerical results are tabulated in the subsections following.

##### a. Heat transfer results

##### Heat transfer enhancement purely via conduction (17 observations)

There is strong indication that nanoparticles can enhance the heat transfer via conduction. All observations indicated an enhancement. Statistically, most observations indicate an enhancement in the range of 10-14% (41% of the sample) with a moderate spread. The performance indication is different to the 5-9% most occurring enhancement regime indicated by level 1 but overall lies in the 1-24% general enhancement regime also indicated by level 1.

*CHF enhancement (13 observations)*

All observations indicate an enhancement. Most observations show statistically an improvement of the CHF coefficient in the range of 100-200% (46% of the sample). This figure agrees with the performance value of the same heat transfer mode observed in level 1.

##### b. Proposed mechanisms for heat transfer anomalies

*Conduction/convection/mixed mode heat transfer (16 samples)*

The statistics indicate that a large percentage of researchers explain the heat transfer enhancement by the interfacial layer theory and the Brownian motion theory in combination with the thermophoretic effect on nanoparticles (37.5% of the sample for each category). The Brownian motion theory comes third most popular (25% of the sample). Differences are found compared to the findings of the 1st level of analysis concerning the most occurring proposed mechanism for heat transfer anomalies.

*PBHT and CHF enhancement (17 observations)*

The analysis indicates that the majority of researchers (82.4%) account for the heat transfer enhancement through the alteration of the heating surface due to deposition of nanoparticles. The second proposed mechanism is the aggregation and diffusion in combination with the thermal conductivity enhancement alone theories (11.8% of the sample). The third most favoured mechanism refers to the electrical double layer (EDL) in combination with the aggregation and diffusion and the alteration of the heating surface due to the deposition of nanoparticles theories (5.9% of the sample). The most probable mechanism agrees with the findings of the first level however, the second and third most probable mechanisms do not agree with the first analysis level.

#### Simple pipe flow experiments

To investigate the convection/mixed heat transfer mode it was decided to narrow the sample to the experiments involving flow in a heated pipe. Temperature measurements are made and in conjunction with the already established physics governing flow in heated pipes it is possible to extract convective heat transfer performance data. This kind of experiments represents the simplest experimental arrangements around the convective mode performance assessment found in the literature. The outline of the section is similar to the analysis performed for the transient hot-wire experiments. No histograms are present but instead the results are presented in their numerical form.

##### a. Heat transfer results

*Heat transfer enhancement via conduction (19 observations)*

There is strong indication that nanofluids can enhance the conductive heat transfer mode. All observations indicated an enhancement. Most observations indicate a heat transfer enhancement in the range 5-9% (42% of the sample). This performance value agrees with the one found in analysis level 1.

*Heat transfer enhancement via convection/mixed heat transfer mode (28 observations)*

The majority of the publications shows heat transfer enhancement in the range of 10-14% (14% of the sample). However, the spread of the enhancement results is large. There is also moderate statistical evidence that the addition of nanoparticles does not change the thermal performance of the heat transfer via convection/mixed mode (36% of the sample). The findings agree partially with the ones found in level 1 (level 1 produced a probable enhancement of 10-19%).

##### b. Rheological studies (23 observations)

There is strong indication that nanoparticles enhance the effective viscosity of the considered nanofluids (78% of the sample indicates an increase). Most publications have on average a viscosity enhancement between 5 and 14% (34% of the sample) and moderate evidence showing that the addition of nanoparticles has no effect on the effective viscosity of nanofluids (22% of the sample). The rheological studies of this section contain the vast majority of experiments of this kind in the entire observation sample hence a comparison with level 1 cannot be performed.

##### c. Proposed mechanisms for the heat transfer anomalies

*Conduction/convection/mixed mode heat transfer studies (13 observations)*

The statistics indicate that the majority of researchers explain the enhancement of heat transfer through the Brownian motion of nanoparticles in combination with the aggregation and diffusion theory (53.8% of the sample), followed by the second most statistically occurring, which is the Brownian motion theory alone (30.8% of the sample). Therefore, the majority of the researchers believe that the Brownian motion is the main mechanism and this is in agreement with the results of level 1.

### Discussion of findings from level 3 analysis

The thermal performance analysis of the narrowed down sample agreed moderately well with the analysis performed in level 2 and the entire population of observations analysed in level 1.

Specifically, for the transient hot-wire experiments, the conduction mode enhancement was found to lie close to the arithmetical values found in level 1 and level 2, while, for CHF, the enhancement was also found in agreement with the previous two levels of analysis. For conduction/convection/mixed heat transfer, the most popular proposed mechanisms are in relatively good agreement with those of level 1 and level 2 (the two most probable mechanisms are followed by a third-the thermophoretic effect theory). The deposition of nanoparticles on the heating surface was identified as the most popular mechanism in explaining the heat transfer anomalies for PBHT and CHF, which is also in good agreement with the findings of level 1 and level 2 analyses.

For the simple pipe flow experiments, the heat transfer enhancement via pure conduction and convection/mixed modes were in good agreement with those of level 1 and level 2 analyses. The most popular mechanism to explain the observations was the Augmented Brownian motion in combination with the aggregation and diffusion mechanism, which again are in good agreement with the results of the level 1 and level 2 analyses.

Apart from the examination of the heat transfer performance, the current authors examined the correlation between several controlling parameters and nanofluid thermal performance in order to investigate if the trends were the same as those of level 2 analysis. The five parameters under investigation were the nanofluid type (basefluid and nanoparticle materials), the nanoparticle size and concentration along with the flow type (stationary/turbulent/laminar flow) and nanofluid temperature. The analysis yielded no correlation between the parameters associated with this sample even though the sample size appears arithmetically sufficient.

The reason for the absence of any correlation between the observations and their by part properties was further investigated. It was discovered that the combination of the different parameters resulted in a variety of different experimental conditions, thus any comparison of any individual parametric effect was impossible as the sample became too small to study. At the same time, it was possible to indirectly deduce that the investigated five parameters play an important role in the emerging thermal performance of nanofluids. Finally, the 3rd level of analysis pointed out that despite the large sample of collected publications for this literature review (more than 250), no study was performed to take into account simultaneously all five parameters and their effects on the thermal performance of nanofluids.

## Nanoemulsions

Nanoemulsions are a new type of fluids that bear similarities with nanofluids. They emerged recently in the literature and have been attracting attention from the research community. Nanoemulsions usually comprise of an insoluble mixture of droplets with a single or a soluble mixture of fluid(s) as the base/carrier fluid, i.e. fluid-in-fluid mixtures. This is in contrast to the broader definition of the two phase nature of nanofluids (solids-in-fluid mixtures). The most common formation of nanoemulsions arises from a stable suspension of droplets of one of the main constituents into the other(s) at various concentrations where the stability of the emulsion is still ensured.

The droplets have sizes of the nanoscale order and their stability is ensured by means of surfactants (contrary to nanofluids where the stability can also be ensured without the need of a surfactant). This kind of fluids exhibited heat transfer augmentation effects in conduction studies. Thermal enhancement up to 52% was reported upon testing a nanoemulsion mixture of water-in-FC72 oil [129], while experimentation with nanoemulsions of oil in a binary mixture of H_2_O/LiBr [130] and water in *n*-decane [131] indicate augmentations of 3.6% and very little augmentation to suggest any heat transfer potentials, respectively.

One of the most appealing aspects of nanoemulsions is that they can be mass produced using cheap emulsification techniques and the emulsion can hold the carrier fluid's electrical properties while the suspension stability (under specific conditions) can be larger compared to their conventional two phase nanofluid counterparts. One of their major drawbacks is that the heavy use of surfactants to achieve mixture stability leads to hysteresis phenomena in the thermal performance of these fluids. An additional issue-this time to be foreseen from the literature review analysis on nanofluids-is that the stability of the nanoemulsion is probably going to be endangered in high temperature heat transfer as the conventional surfactant chemical composition at those temperatures changes irreversibly (surfactants are destroyed).

All in all, the area of thermal nanoemulsion performance is rather immature to reach to conclusions regarding the thermal performance of such fluids, since not enough experiments have been performed up to date to quantify creditable statistics.

## Summary and future research needs

A literature review was performed, which statistically analysed a large amount of literature regarding the anomalous heat transfer modes exhibited by nanofluids. Three levels of analysis were selected. The first one allowed the extraction of results concerning general heat transfer characteristics and performance of nanofluids. The second one focused on revealing any possible trends linking the heat transfer performance of certain nanofluids with their by part parameters. The third level revealed the parameters that appear to control the thermal performance of nanofluids and indirectly indicated the current research needs to enable reaching a more conclusive result.

All three levels of analysis agreed to a large degree on the choice of proposed mechanisms to explain the anomalous heat transfer, as well as on the thermal performance of nanofluids for different heat transfer modes. This indicates that both diminished sample sets of level 2 and level 3, created from the bulk population of publications, were reliable to proceed with the extraction of statistical results.

The statistical analysis of thermal performance indicated that there is a notable enhancement for conduction, convection/mixed, pool boiling and CHF heat transfer modes. The level of enhancement varies for each sub set with the purely conductive mode showing the least enhancement (around 5-9% most frequently observed), the convection/mixed mode a moderate enhancement (around 10-14%), the pool boiling mode a higher enhancement of 40-44%, while the CHF demonstrated the highest enhancement (100-200%). For some of the considered heat transfer modes, heat transfer coefficient deterioration or no effect were also recorded; however, these occurrences were low.

For the explanation of the enhancement related to conduction, convention/mixed heat transfer modes, the most popular theories revolve around either the Brownian motion of nanoparticles, the interfacial liquid layering (Kapitza resistance) theory, the aggregation and diffusion theory or simply a combination of all three.

For PBHT and the CHF, the most commonly proposed mechanisms are the alteration of the heating surface by deposition of nanoparticles along with the passive/active mode theories.

The sample set of the level 2 analysis indicated that for the purely conductive mode, the basefluid material has negligible effect to the enhancement of heat transfer. It was also possible to define trends, linking properties of nanofluids with their thermal performance as follows:

• The level of enhancement for the purely conductive case indicated an increasing trend with increasing nanofluid temperature and nanoparticle concentration, while there is a slight hint of enhancement with increasing nanoparticle size.

• The level of enhancement for the convection/mixed heat transfer mode indicated an increasing trend with increasing temperature, volumetric concentration and decreasing nanoparticle size.

• The effective viscosity of nanofluids increases with decreasing temperature of the fluid and increasing volumetric concentration. There is also a slight hint of the effective viscosity increase with decreasing nanoparticle size.

The trends remain valid up to the degree where the nanofluid defining qualities (regarding particle suspension and chemical consistency properties as listed in "Characteristics of nanofluids" section) are still satisfied and the nanoparticle concentrations remain in between the boundaries set in the methodology of observation collection (0.0001-10 vol.%). The most popular mechanisms proposed in the literature to explain the heat transfer anomalies observed supported the trend behaviour observed.

Based on the findings of the statistical analysis of the literature, some recommendations for future research are provided below.

(a) The final level of analysis, level 3, outlined the five apparent parameters controlling thermal performance. Those are the nanofluid type (Basefluid and nanoparticle materials), the nanoparticle size and concentration, the flow type (stationary/turbulent/laminar flow) and lastly the nanofluid temperature. Level 3 analysis indicated that no parametric study has been performed that takes into account all five parameters noted above to evaluate their contribution on the thermal performance of nanofluids.

(b) The review demonstrated that, even though attempts were frequently made to ensure that the quality criteria of the prepared nanofluids were satisfied prior the experiments, no attempt has been presented to re-examine the nanofluids after or during experiments-with the exceptions of some cases of pool boiling or CHF investigations or microchannel clogging investigations due to nanofluids. As a result, it is doubtful whether the nanofluid properties (e.g. nanoparticle size) remain the same during or after experiments.

(c) No study has been performed to quantify any corrosion effects during long term operation of nanofluids heat/cooling circuits.

(d) The mechanism responsible for the observed heat transfer enhancement has not been verified experimentally. This is an important step for the optimisation of the performance of nanofluids and the development of appropriate computational models to describe their behaviour.

(e) A larger amount of experimental studies are related to conduction and less is available for convection, pool boiling and CHF modes. Therefore, more emphasis should be given to these modes of heat transfer in future experimentation.

Finally, a brief consideration of a new type of fluid that bears similarities to nanofluids and might in the future be part of the broader nanofluid category has been examined. nanoemulsions are single-phase liquid-into-liquid mixtures that can potentially be of interest due to their abnormal thermal performance. Unfortunately, not enough experiments have been performed to allow statistical analysis of their performance.

## Abbreviations

BET: Brunnauer-Emmet-Teller; CNT: carbon nanotube; CHF: critical heat flux; DLS: dynamic light scattering; EDL: electrical double layer; EG: ethylene glycol; HT: heat transfer; NF: nanofluids; PBHT: pool boiling heat transfer; SEM: scanning electron microscopy; TEM: transfer electron microscopy.

List of symbols

A = 

when ellipsoid is defined by

*a*, *b *and *c *are constants indicating the points where the ellipsoid is crossing the *x*, *y *and *z*-axis, respectively CHF: Critical Heat Flux

*f*: volume concentration of the solid ellipsoids without surrounding layers; f_e_= r _v_f; HT: Heat transfer; K: heat transfer coefficient; k_pe_: equivalent thermal conductivity of equivalent Nanoparticle; k_pj_: equivalent thermal conductivities along the axes of the complex ellipsoid

NF: Nanofluids; PBHT: Pool Boiling Heat Transfer; r_v_: volume ratio of nanolayer to nanoparticle

Subscripts

BF: Basefluid (host fluid); eff: Effective; f: Fluid; NF: Nanofluid; NP: Nanoparticle; p: Solid particle

Greek symbols

ρ: density; Φ: volumetric concentration; Φ_m_: mass concentration; ψ: sphericity

## Competing interests

The authors declare that they have no competing interests.

## Authors' contributions

AS conceived the study and performed the statistical analysis. YH participated in the design and coordination of the study. All authors read and approved the final manuscript.
